# Carbon cycle instability and orbital forcing during the Middle Eocene Climatic Optimum

**DOI:** 10.1038/s41598-019-45763-2

**Published:** 2019-06-27

**Authors:** Martino Giorgioni, Luigi Jovane, Eric S. Rego, Daniel Rodelli, Fabrizio Frontalini, Rodolfo Coccioni, Rita Catanzariti, Ercan Özcan

**Affiliations:** 1 Instituto Oceanográfico, Universidade de São Paulo, São Paulo, 05508-120 Brazil; 2 Dipartimento di Scienze Pure e Applicate (DiSPeA), Università degli Studi di Urbino “Carlo Bo”, 61029 Urbino, Italy; 3 Istituto di Geoscienze e Georisorse, Consiglio Nazionale delle Ricerche (CNR), 56124 Pisa, Italy; 4 Faculty of Mines, Department of Geological Engineering, Istanbul Technical University (ITU), Maslak, 34469 Istanbul Turkey; 5 Present Address: Instituto de Geociências, Universidade de Brasília, Brasília, 70910-900 Brazil; 6 Present Address: Instituto de Geociências, Universidade de São Paulo, São Paulo, 05508-080 Brazil

**Keywords:** Carbon cycle, Palaeoceanography, Palaeoclimate

## Abstract

The Middle Eocene Climatic Optimum (MECO) is a global warming event that occurred at about 40 Ma. In comparison to the most known global warming events of the Paleogene, the MECO has some peculiar features that make its interpretation controversial. The main peculiarities of the MECO are a duration of ~500 kyr and a carbon isotope signature that varies from site to site. Here we present new carbon and oxygen stable isotopes records (δ^13^C and δ^18^O) from three foraminiferal genera dwelling at different depths throughout the water column and the sea bottom during the middle Eocene, from eastern Turkey. We document that the MECO is related to major oceanographic and climatic changes in the Neo-Tethys and also in other oceanic basins. The carbon isotope signature of the MECO is difficult to interpret because it is highly variable from site to site. We hypothesize that such δ^13^C signature indicates highly unstable oceanographic and carbon cycle conditions, which may have been forced by the coincidence between a 400 kyr and a 2.4 Myr orbital eccentricity minimum. Such forcing has been also suggested for the Cretaceous Oceanic Anoxic Events, which resemble the MECO event more than the Cenozoic hyperthermals.

## Introduction

The Middle Eocene Climatic Optimum (MECO) is a global warming event, which occurred at about 40 Ma and lasted ~500 kyr. It is characterized by a decrease in oxygen isotope (δ^18^O) values, suggesting up to 6 °C warming of the sea surface water^1,2^. It is also related to a widespread carbonate decrease in pelagic settings, attributed to seawater acidification, which induced shallowing of the lysocline and of the carbonate compensation depth (CCD), like in the Paleocene Eocene Thermal Maximum (PETM) and the other hyperthermals (e.g. ETM2 and ETM3)^1,3,4^. However, the MECO cannot be easily interpreted as one of these events.

Transient climate warming events are commonly explained by the rapid intensification of the greenhouse effect. This is supported by a prominent negative carbon isotope (δ^13^C) peak occurring at the onset of these events in response of massive dissociation of gas hydrates or extensive volcanic events that injected large amounts of light carbon into the ocean-atmosphere system^5–8^. The effects of these phenomena at the ten thousand-years’ time scale are warming and seawater acidification induced by the increasing *p*CO_2_^6^. However, over hundreds of thousand years, such as the duration of the MECO, high atmospheric *p*CO_2_ increases the weathering of surface rocks, which draws *p*CO_2_ from the atmosphere and increases the seawater alkalinity. This means that at the time scale of the MECO, a massive injection of greenhouse gases into the atmosphere and the ocean is counterbalanced by carbon cycle feedbacks and would produce an increase in deep-water carbonate deposition, instead of a decrease^9,10^.

Another important feature of the MECO is that it does not show a distinct δ^13^C negative peak, which is, instead, a diagnostic feature of the PETM and other hyperthermals. Negative δ^13^C excursions occur only in some of the records of the MECO and not always at the onset of the warming conditions^2,11^. Such isotopic signature, alongside the duration, suggests that the MECO was triggered by different factors from the transient warming events.

Here we present a new stable isotopes record of the MECO event from eastern Turkey. Our data are from three foraminiferal genera dwelling at different depths throughout the water column, showing signals from the bottom, thermocline, and surface. Our results show that the warming peak of the MECO is slightly preceded by a negative δ^13^C anomaly, but it is also followed by a δ^13^C increase. By comparison with isotopic records from other oceanic basins we show that the MECO is characterized by high carbon cycle instability and occurs soon after a minimum in both 400 kyr and 2.4 Myr eccentricities, suggesting a possible causal link. All evidence indicates that the MECO was different from the Cenozoic hyperthermals, but had several features in common with the Oceanic Anoxic Events of the Cretaceous.

## Results

### Carbon and oxygen stable isotopes from the Baskil section

**Figure 1:**
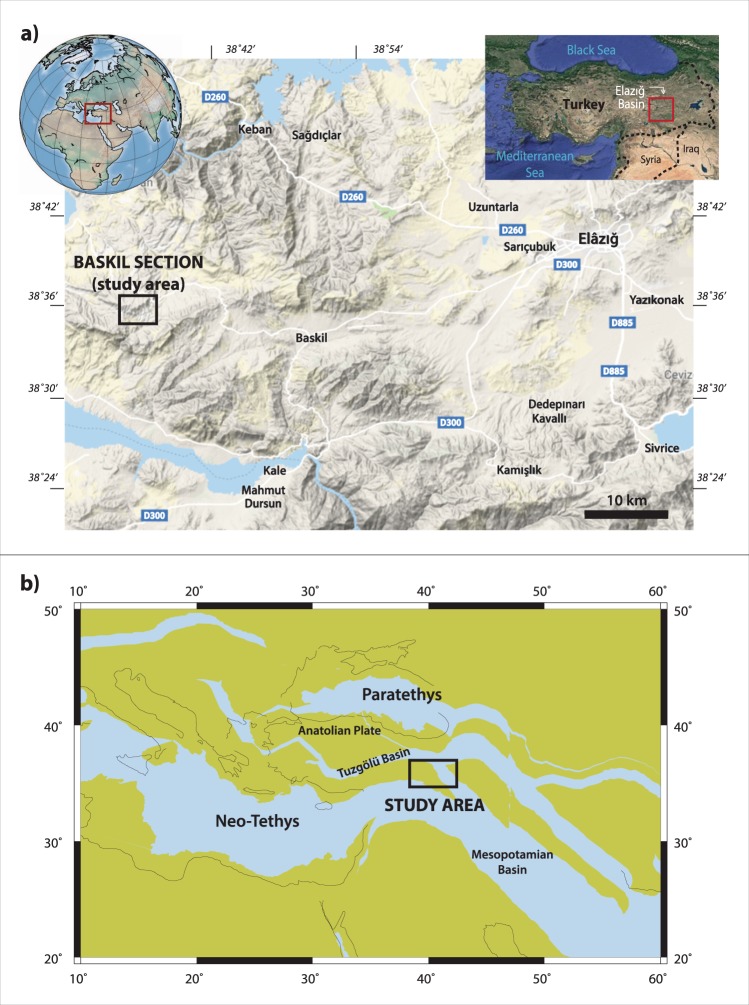
(**a**) Geographic maps showing the location of the studied area. The base of the terrain map is from Google Maps (Map data©2019 Google; https://www.google.com/maps/@38.6360375,38.9277941,10z/data=!5m1!1e4); the satellite map of Turkey at the top right corner is from Google Earth (Map data©2018 Google; https://www.google.com/maps/); the globe at the top left corner was made using Python 3.4.3-0 with Matplotlib^59^. (**b**) Paleogeographic map of the eastern part of the Neo Tethys at 40 Ma, showing the paleogeographic location of the studied area (generated with the ODSN Plate Tectonic Reconstruction Service, 2011; http://www.odsn.de/odsn/services/paleomap/paleomap.html).

The Baskil section consists of a mixed carbonate-siliciclastic succession encompassing a highly continuous and expanded stratigraphic record of the Bartonian stage (41.2–37.8 Ma) in eastern Turkey (Fig. 1). This section was dated with various well-preserved groups of microfossils, such as planktonic foraminifera, calcareous nannofossils, and large benthic foraminifera, as well as magnetostratigraphy^12^.

**Figure 2:**
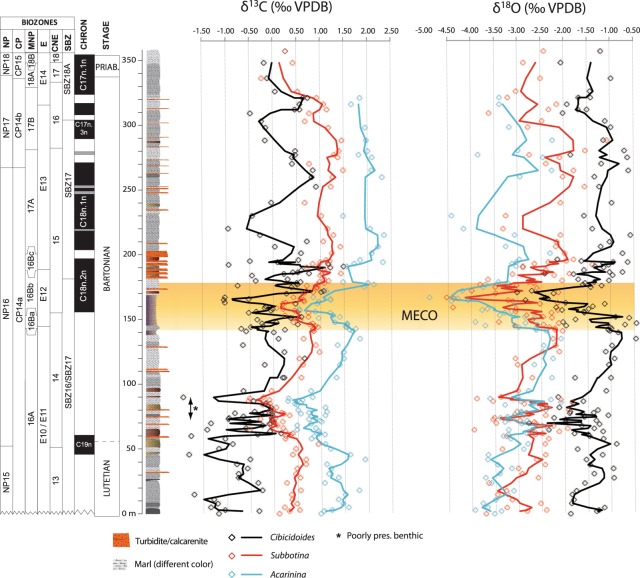
Magnetobiostratigraphy, lithological log, and stable isotopes curves of the Baskil section. Litho-, magneto-, and biostratigraphic data are from ref.^12^. Carbon (δ^13^C) and oxygen (δ^18^O) isotopes are shown for three individual genera of foraminifera, representing surface (*Acarinina*), thermocline (*Subbotina*) and bottom (*Cibicidoides*) conditions. Diamonds represent individual data points, whereas solid lines represent curves smoothed by 3pts moving average. The yellow shaded bar highlights the position of the MECO based on its isotopic signatures.

**Figure 3:**
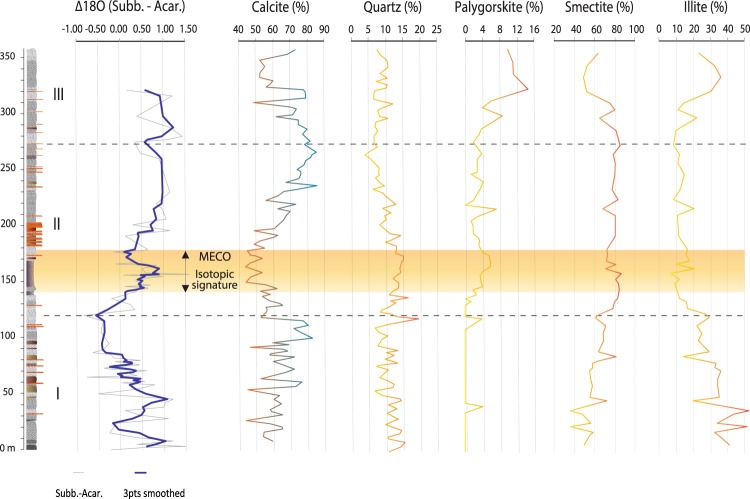
∆^18^O and abundance of calcite, quartz, palygorskite, smectite and illite throughout the Baskil section. ∆^18^O is the difference between the δ^18^O measured on *Subbotina* and *Acarinina*, and is related to the temperature difference between the sea surface and the thermocline. The thin grey line represents the raw ∆^18^O curve, whereas the thick blue line represents the 3pts moving average smoothed curve. Minerals abundance is from ref.^16^, note that quartz and calcite are respect to bulk minerals abundance, whereas clays are respect to bulk phyllosilicates abundance. The intervals I, II, and III represent the climatic phases described in ref.^16^. The yellow shaded bar highlights the position of the MECO isotopic signatures shown in Fig. 2.

Carbon and oxygen stable isotopes were measured on bulk carbonate and on selected foraminiferal genera (Fig. 2). The bulk carbonate isotopic records show variations that may be due to different carbonate sources, and therefore are not considered for paleoclimatic interpretation (see Supplementary Information). Stable isotopes were measured on *Cibicidoides* (benthic), *Subbotina* (thermocline dwelling), and *Acarinina* (surface dwelling) foraminiferal genera (Fig. 2). *Acarinina* and *Subbotina* δ^13^C curves show the same trend, although the former has higher values than the latter. The curves decrease gradually to a minimum at 86 m and then increase by 1.5‰ towards the upper part of the section. This increasing trend is interrupted by a negative peak of ~1‰ between 142 m and 178 m. A similar trend also occurs in the benthic foraminifera δ^13^C curve, although with lower values and a noisier pattern overall (Fig. 2). Oxygen isotope curves have generally the lightest values in *Acarinina*, slightly heavier in *Subbotina* and the heaviest in *Cibicidoides*. However, they show similar trends, particularly a prominent negative peak of 1.5–2.0‰ at 10 m above the negative δ^13^C peak. In the lower part of the section, up to 140 m, the curves of the surface and deep dwelling planktonic foraminifera almost overlap, with a divergence of ~1‰ at around 42 m. From 140 m upwards, the two curves begin to diverge and reach a difference of 1‰ in the upper part of the section. This divergence is represented as ∆^18^O, which is calculated as follows:

1 $${{\rm{\Delta }}}^{18}{\rm{O}}={{\rm{\delta }}}^{18}{{\rm{O}}}_{{\rm{subb}}{\rm{.}}}-{{\rm{\delta }}}^{18}{{\rm{O}}}_{{\rm{acar}}.}$$

where δ^18^O_subb._ and δ^18^O_acar._ are the δ^18^O values of the genera *Subbotina* and *Acarinina*, respectively (Fig. 3).

### Significance of the geochemical record of the Baskil section

Scanning electron microscope (SEM) and data analyses on representative specimens of the three studied foraminiferal genera show that significant post-depositional alteration of the isotopic composition can be excluded and variations larger than 0.5‰ can be interpreted as due to changes in environmental conditions (see Supplementary Information). The difference in values between the δ^13^C curves of the three foraminiferal genera reflects the difference in isotopic composition of the dissolved inorganic carbon (DIC) in their original living environments. More intense biotic activity at the surface produces faster carbon recycling and therefore higher δ^13^C values, as observed in the surface dwelling *Acarinina*. The amount of light carbon released by the remineralization of organic matter increases closer to the thermocline and even more at the bottom, explaining the lower δ^13^C values of *Subbotina* and the even lower of *Cibicidoides*. Similar considerations can be made on δ^18^O values, which are mainly related to the seawater temperature (see Supplementary Information). The difference between the δ^18^O curves reflects the temperature gradient throughout the water column, which is warmest at the surface, cooler at the thermocline, and coolest at the bottom. This is expressed by the lightest δ^18^O of *Acarinina*, heavier δ^18^O of *Subbotina*, and the heaviest δ^18^O of *Cibicidoides*, respectively. The prominent negative peaks in δ^13^C and δ^18^O occurring between 142 m and 178 m correspond to the planktonic foraminiferal biozone E12 (*Orbulinoides beckmanii*), the calcareous nannoplankton subzones MNP16Ba-MNP16Bb, and the boundary between the C18r and C18n magnetochrons, constraining the δ^13^C and δ^18^O signatures to the MECO event^1,2,13^. The stable isotope records of the Baskil section confirm that significant warming occurred during the MECO and that this affected the entire oceanic system, as its signature is recorded in foraminifera living in shallow, deep, and bottom environments. Moreover, the planktonic and benthic foraminiferal stable isotope records do not exhibit any time lag, implying that the oceanic mixing was faster than the change in the oceanic isotopic reservoir, differently from abrupt transient events^8^. The ∆^18^O represents the temperature gradient between the sea surface and the thermocline, which is related to the degree of mixing of the seawater. Thus, the increase in ∆^18^O starting at 120 m may reflect enhancing stratification of the upper part of the water column, which induces a steeper temperature gradient (Fig. 3). Another explanation for such ∆^18^O increase is that the thermocline dwelling *Subbotina* adjusted its calcification depths in response to the warming conditions, possibly following isotherms or the nutricline, whereas the vertical motion of *Acarinina* could be limited only to the euphotic zone due to its photosymbionts^14^. Migration of foraminiferal taxa through the thermocline can bias the ∆^18^O, which would record a change in habitat rather than in the depth of the thermocline^15^. However, in the Baskil section, the increase in ∆^18^O is also accompanied by changes in mineralogical and calcareous nannofossils assemblages, suggesting a major rearrangement in the water column rather than a shift of the habitat (Figs 3 and S3)^16^.

A shift towards warm and humid climatic conditions was identified in the MECO interval of the Baskil section^16^ as inferred by the relative increase in the abundance of smectite and parallel decrease in illite (Fig. 3). These conditions increased the amount of detrital input that diluted, and probably also limited, the carbonate production (Fig. 3). Such conditions could have freshened the sea surface and favoured a more stratified water column in the Neo-Tethys by increasing the density contrast. Moreover, the occurrence of authigenic palygorskite suggests the ingression of a new bottom water mass that delivered Mg into the basin. The formation of palygorskite is coincident with the onset of MECO but was enhanced after the end of this event, when the climatic conditions became dryer (Fig. 3)^16^.

## Discussion

**Figure 4:**
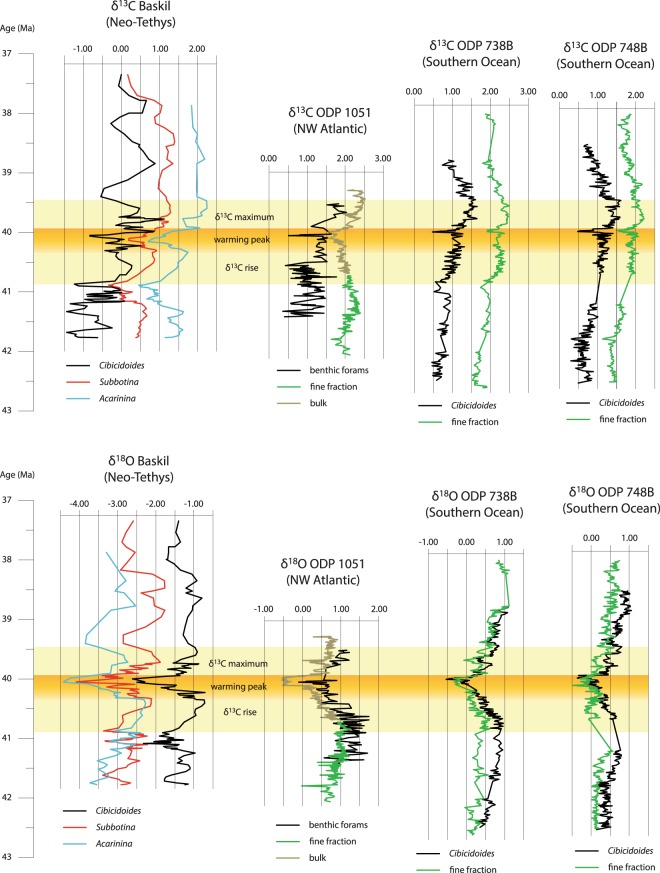
Bottom and upper water column carbon and oxygen stable isotopes records from the middle Eocene. The lower pale yellow bar highlights where the δ^13^C starts increasing, corresponding also to the onset of the warming in the Southern Ocean. The dark yellow bar highlights the MECO warming peak, defined as the negative δ^18^O anomaly at around 40 Ma. The upper pale yellow bar highlights the interval with highest δ^13^C values. Data are from this study and refs^2,13^ (see methods for more details).

A comparison between the Baskil section and other middle Eocene isotopic records representing the bottom and upper parts of the water column is provided in Fig. 4. The Baskil section displays values that are, on average, 1.0‰ to 1.5‰ lower for δ^13^C and 2.0‰ to 4.0‰ lower for δ^18^O, and the amplitude of the fluctuations is generally larger in the Baskil section than in the other records (Fig. 4). Moreover, the difference in δ^18^O values between the bottom and the upper water column counterparts is much larger in the Baskil section. These differences can be attributed to the depositional setting, as the other records are from relatively open pelagic environments, whereas the Baskil section formed in relatively restricted hemipelagic conditions^2,12,13^. The Neo-Tethys in the middle Eocene was a narrow basin with a complex topography, similar to the Mediterranean Sea^17^. Therefore, the water column was highly stratified and the continental runoff delivered significant amounts of light carbon and fresh water, which lowered the δ^13^C and the δ^18^O of the seawater, respectively. Consequently, the isotopic fluctuations were controlled in part by local factors and a relatively small size reservoir^18^. A more stratified water column with fresh water input could have lowered the δ^18^O at the surface, so the ∆^18^O increase may be attributed to a combined signal of both temperature and salinity. However, the fact the amplitude of the negative δ^18^O anomaly is the same in the surface, thermocline, and bottom dwelling foraminiferal genera indicates that temperature was likely the main controlling factor. Moreover, considering the tectonic history of the basin, burial diagenesis might also have further decreased the general isotopic values, though in minor extent (see Supplementary Information).

Despite the differences in absolute values, the isotopic signature of the Baskil section correlates clearly with pre-existing records of the middle Eocene and provides new insights into the MECO event. In the Baskil section, the δ^18^O starts decreasing at 40.3 Ma, reaches its minimum peak at 40.0 Ma, and then increases back to pre-decreasing values at 39.7 Ma (Fig. 4). This indicates that the MECO warming anomaly in the Neo-Tethys lasted about 600 kyr, which is similar to the estimates from previous studies^2,13,19,20^. However, when compared to other records from the Southern Ocean and the NW Atlantic, it seems that this isotopic anomaly represents only the last stage of a warming trend. This is also accompanied by an increasing trend of δ^13^C, interrupted by a prominent negative spike that occurs at 40.1 Ma at Baskil, and about 100 kyr later in the Southern Ocean. On the other hand, the δ^13^C signal in the NW Atlantic is different and displays a gradually decreasing trend of 0.8‰, reaching a minimum at 40.1 Ma and then increasing by about 1.0‰ till 39.4 Ma (Fig. 4). This evidence raises questions about the actual duration and nature of the MECO event. Is the ~600 kyr warming peak the most extreme phase of a ~1.2 Myr global warming event, or is it just superimposed onto a longer-term climatic fluctuation? Which carbon cycle mechanisms can produce such different signals at the hundred thousand-years time scale?

The origin of the MECO is a highly controversial issue in current paleoclimate science^9^. Our results from the Baskil section reveal that it is related to changes in paleocirculation in the Neo-Tethys and a concomitant shift towards warmer and wetter climatic conditions at regional scale^16^ (Fig. 3). Changes in paleocirculation and paleoproductivity during the MECO have been inferred also in various other sites. In most cases, increased primary productivity associated to stronger currents and acceleration of the biological pump was observed^20–26^. In other sites, high primary productivity was interpreted as stimulated by increasing aeolian dust, suggesting dry climatic conditions^27–31^. Continental records indicate a shift towards hot and humid conditions in North America, similar to Turkey^32,33^, but also dry conditions in central Asia^34^. Such different climatic conditions at regional scale suggest weakening of the major climatic forcings during the MECO warming phase.

**Figure 5:**
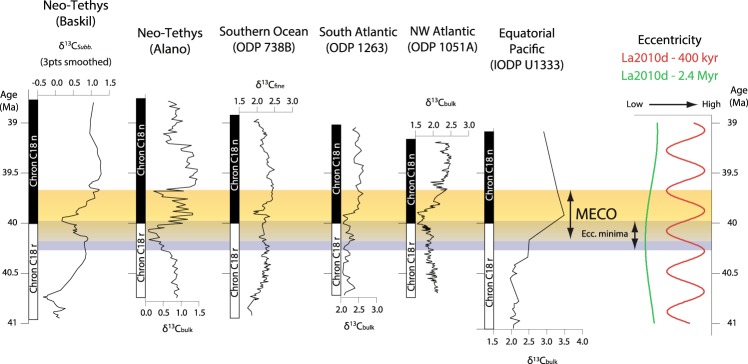
Middle Eocene carbon isotope curves from six representative regions, correlated according to the base of the magnetochron C18n and calibrated respect to the age model of ref.^39^. The yellow bar highlights the position of the MECO and the grey one the co-occurring minima of 400 kyr and 2.4 Myr eccentricity cycles. Data are from this study, and refs^2,17,31,51,52^ (see methods for more details).

Another indication of such variable climatic conditions can be the “undefined” carbon isotope signature, indicating high carbon cycle instability during the MECO (Fig. 5). The δ^13^C records of the MECO in Fig. 5 are the most complete and representative of the various oceanic basins, and have been correlated according to the base of the magnetochron C18n. All of them display some variation within the MECO interval, but there is no distinct signature. Negative peaks occur only in some of the records, and with variable amplitude and stratigraphic position. After such highly variable interval, all the curves display a δ^13^C increase of 0.5‰ to 1.0‰, which is the most consistent feature in all the records. Such undefined carbon isotope signature expresses a rather unstable oceanic carbon cycle over hundreds of kyrs during the MECO. It is also worth noting that the δ^13^C rise starting at around 41 Ma, observed in the Baskil and ODP Site 738 curves (Fig. 4), is not expressed in other regions. This suggests that the MECO is an event of enhanced climatic instability occurring within a longer-term unstable climatic phase during the middle Eocene.

The carbon cycle instability recorded during the MECO is difficult to explain because it has a “mid-term” duration (10^5^ yr), meaning that it is too long for being comparable with rapid transient events (10^4^ yr), and too short for being considered a long-term event, driven by tectonic and paleogeographic changes (≥10^6^ yr). For this reason, possible triggers must be searched amongst those factors that act at the hundred thousand year-time scales. Volcanism can be a possibility, but there is no reported evidence of sufficiently intense volcanic activity near the onset of the MECO. Another possible trigger can be Milankovitch orbital eccentricity, which is able to produce isotopic variations with the amplitude of those occurring during the MECO^35–37^. An orbital forcing for the MECO has been proposed by ref.^38^, who found that the MECO occurs at two coinciding minima of 400 kyr and 2.4 Myr eccentricity cycles. Here we recalibrate the age of the MECO respect to the most updated age model by ref.^39^, which sets the base of the magnetochron C18n at 40.0 Ma. According to this new calibration, the δ^13^C signature of the MECO can be considered the most variable part of the curves between 40.2 Ma and 39.7 Ma, starting with a negative shift in some places and ending with a δ^13^C maximum. This signature occurs soon after a knot between a 400 kyr and 2.4 Myr eccentricity minima (Fig. 5). This suggests that such specific orbital configuration forced the strong carbon cycle instability during the MECO.

It still remains to explain how a prolonged low orbital eccentricity might have induced climate warming and carbon cycle instability. Although the precise mechanisms are not clear, it has been shown that such orbital configuration occurs also related to the Oceanic Anoxic Events (OAEs) in the Late Cretaceous^40,41^. OAEs are episodes of widespread oxygen depletion in deep-water settings that occurred mainly during the Cretaceous and had durations of few to several hundreds of kyr. They are related to intense climatic and carbon cycle perturbations, which are considered produced by massive injections of CO_2_ into the atmosphere, derived from large volcanic provinces^42–45^. However, refs^40,41^ identified a possible coincidence between 400 kyr and 2.4 Myr low eccentricities and the OAE 2, at the Cenomanian-Turonian boundary (~94 Ma), and proposed that orbital forcing could have enhanced the effect of the volcanic trigger. Moreover, ref.^41^ detected also the obliquity signal within this interval, and therefore suggested that the extremely low eccentricity produced a prolonged period with a relatively more stagnant ocean and low seasonal contrast, during which the climate was more variable at the shorter-term. These conditions could have occurred also during the MECO and may explain the highly variable δ^13^C records. On the other hand, it is worth noting that the one at around 40 Ma is not the only knot between 400 kyr and 2.4 Myr low eccentricities occurring during the Eocene^46^. This indicates that orbital forcing gave the final pulse to trigger climate warming and carbon cycle instability in particular boundary conditions that enhanced the sensitivity during the middle Eocene. Such conditions could be related to the longer-term oceanographic modifications in the Southern Ocean, induced by the opening of the Drake Passage and the Tasman Gateway^47,48^, or in the Tethys, due to the convergence and beginning of the collision between India and Asia^49^.

A climatic scenario with weak seasonal contrast and slow hydrological cycle at the 10^5^ yr time scale is compatible with the decreasing global weathering rate indicated by Os isotope records during the MECO^50^. On the other hand, due to the unstable climate and carbon cycle, episodes of wetter conditions with enhanced chemical weathering and runoff could occur at regional or local scale^16,20,33^. It is interesting to note also that the most consistent feature occurring in the δ^13^C records of the MECO is an increasing trend following the highly unstable interval (Figs 4 and 5). This resembles, to some extent, the δ^13^C signatures of the OAEs, which represent the light carbon sequestration due to the widespread burial of organic matter^42^ and references therein. Particularly, the carbon isotope anomalies of the MECO have amplitude and duration similar to the minor OAEs, such as the OAE 1d. This event lasted a few hundreds of kyr and is characterized by a δ^13^C increase of 0.5‰ to 1.0‰, preceded by a more variable interval, in some cases displaying abrupt negative shifts^51–54^. Besides, also this event could have been related to exceptionally low eccentricity^40^. Although there is very little evidence of widespread organic carbon burial during the MECO^11^, it is worth noting that in terms of duration and isotopic signature it is more similar to an OAE than a hyperthermal.

## Methods

Stable isotopes were measured in the Stable Isotopes Laboratory at the Geoscience Institute of the University of São Paulo (Brazil), using a Thermo Scientific Delta V isotope ratio mass spectrometer coupled in continuous He flow with a chromatographic column included in a GasBench II preparation device. A total of 154 samples of the Baskil section were powdered and measured for bulk carbonate δ^13^C and δ^18^O. As the lithology contains a variable amount of carbonate, there may be variations in the isotopic composition related to different carbonate content that can bias the primary signal. For this reason, 15 samples with different carbonate content were measured in triplicates with different amount of material to check on the variability due to the inhomogeneous carbonate content. Moreover, 7 samples were measured repeatedly in multiple runs to check on possible deviation due to the sensitivity of the measuring instrument. It resulted that both the δ^13^C and the δ^18^O variation amongst each sample triplicates is ~0.4‰, while that between different runs is <0.1‰, which is in the range of the instrumental precision. Thus, in the bulk isotopic records of the Baskil section only variations larger than 0.4‰ are considered for interpretation.

Samples for micropaleontological analyses were prepared following the cold acetolyse technique. This technique enables the extraction of generally easily identifiable foraminifera even from indurated limestones. Specimens of foraminifera of the genera *Cibicidoides*, *Subbotina*, and *Acarinina* were extracted from the washed residue of 125, 123, and 94 samples, respectively. When possible, specimens of all the three genera were extracted from the same sample, however, in some cases, only one or two of the genera were present. Especially, *Acarinina* was consistently absent in the upper part of the section.

The number of specimens picked for stable isotopes analyses depended on the size, as genera with larger shell require lower number of specimens, however, at least 3 specimens per sample were used in order to minimize the vital effect. Five to eight specimens of 250–355 µm were used for *Cibicidoides*, three to five specimens of 300–450 µm for *Subbotina*, and ten to twenty specimens of 150–250 µm for *Acarinina* SEM imaging was done on selected specimens representative for the three studied genera in the Laboratory of Micropaleontology of the University of Brasilia (Brazil). The preservation of the tests was assessed qualitatively under optical microscope and SEM and only the best-preserved specimens were picked for stable isotopes analysis. Moreover, stable isotopes results were checked for anomalies in samples with specimens with slightly poorer preservation than the average. Only 1 result was discarded for having significantly anomalous δ^13^C and δ^18^O values.

Carbon and oxygen isotopes data in Fig. 4 are from the Neo-Tethys (Baskil, this study); the ODP site 1051, bulk and fine fraction^2^, and benthic foraminifera^13^; the ODP sites 738B and 748B, benthic foraminifera and fine fraction^2^.

Carbon isotope data in Fig. 5 are from the Baskil section (this study); the Alano section^21^; the ODP sites 738B, 1263, and 1051 A^2^; the IODP site U1333 (isotopes data by ref.^55^ and age model by ref.^56^). Note that the curve from Turkey is from thermocline dwelling foraminifera (genus *Subbotina*) and has been smoothed by 3pts moving average; all the other curves are from bulk or fine fraction carbonate data. The curves were correlated according to the base of the magnetochron C18n, as identified in each site. The age of this datum was re-calibrated respect to the new age model for the middle Eocene by ref.^39^, and then correlated to the eccentricity curve La2010d^57^ (updated in March 2011), filtered for the 400 kyr and 2.4 Myr periodicities. The filtering was done with AnalySeries 2.0.4.2^58^.

## Supplementary information

Supplementary information for: Carbon cycle instability and orbital forcing during the Middle Eocene Climatic Optimum

## Data Availability

All data from the Baskil section can be obtained from the corresponding author upon request. Data from other sections and already published must be requested from the respective source.
