# Supplementary material for: Carbon cycle instability and orbital forcing during the Middle Eocene Climatic Optimum
Source: Sci Rep. 2019 Jun 27;9:9357. doi: 10.1038/s41598-019-45763-2 (PMC6597698; doi:10.1038/s41598-019-45763-2)
Supplement: Supplementary file 1 — Supplementary information for: Carbon cycle instability and orbital forcing during the Middle Eocene Climatic Optimum [file 41598_2019_45763_MOESM1_ESM.pdf]

Supplementary information for:

Carbon cycle instability and orbital forcing during the Middle Eocene Climatic Optimum

Martino Giorgioni *, Luigi Jovane, Eric S. Rego, Daniel Rodelli, Fabrizio Frontalini, Rodolfo Coccioni, Rita Catanzariti, Ercan Özcan

* Corresponding author: gmartino@unb.br

1) Significance of bulk $\delta^{13}C$ and $\delta^{18}O$ data

The Baskil section deposited at about 400 m depths, in a back-arc basin developed on continental crust during the closure of the Neo-Tethys (Rodelli et al., 2018; Rego et al., 2018). It consists of hemipelagic marls intercalated by coarser carbonate-siliciclastic beds with turbiditic facies. In this context, the main carbonate lithological constituents come both from the nearby neritic carbonate platforms and the settling of the planktonic carbonate producers, living in the pelagic realm. As each of these constituents carries the isotopic signature of its forming environment, the bulk carbonate isotopic composition of the Baskil section reflects a mixture of different carbonate sources rather than variations in the water dissolved inorganic carbon (DIC). Another possible alteration of the stable isotopes signal is diagenesis (e.g. Hudson, 1977). Despite the region underwent syn- and post-sedimentary deformation, the Baskil section remained exceptionally preserved, which excludes the isotopic alteration of deep burial diagenesis and explains the very good preservation of the

occurring nanno-, micro- and macrofossils (Rodelli et al., 2018). However, a possible contribution of early burial diagenesis cannot be excluded.

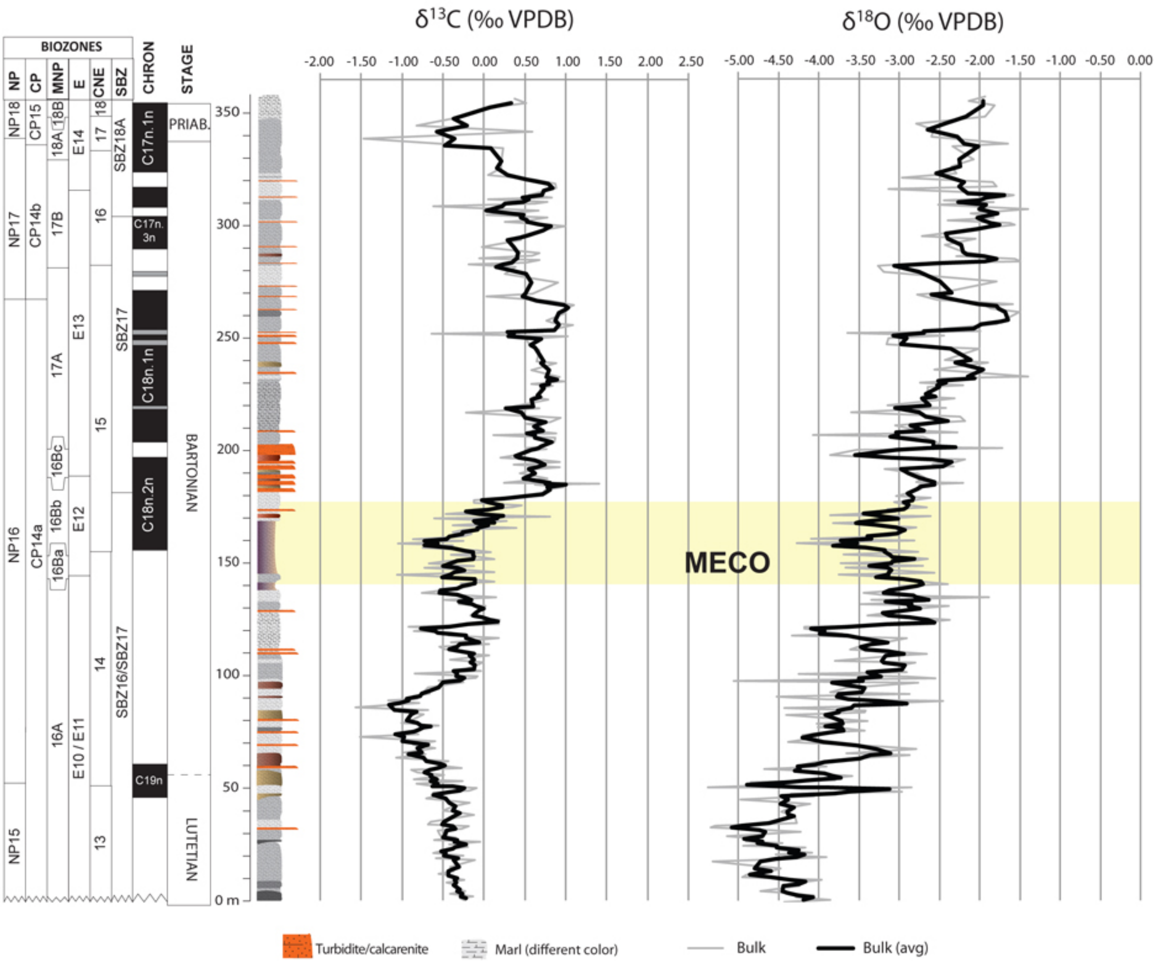

Fig. S1: Bulk carbonate $\delta^{13}C$ and $\delta^{18}O$ curves of the Baskil section, plotted as raw values and smoothed by 3pts moving average.

In order to exclude variations due to the difference in lithofacies, bulk stable isotopes measurements of the Baskil section were only performed on the hemipelagic marl. The bulk carbonate $\delta^{13}C$ curve has highly fluctuating values, with 0.5‰-1.0‰ variations between different lithological beds (Fig. S1). According to the previous considerations, these rapid variations mainly reflect different carbonate sources or possible early burial diagenesis. However, there are more gradual variations that occur consistently throughout different lithological beds. Thus, despite the noise of

the rapid fluctuations, it can be recognized an average trend that can be attributed to secular changes in the marine reservoir of dissolved inorganic carbon (DIC). The same interpretation can be applied to the $\delta^{18}O$, probably with a more significant contribution of diagenesis in the rapid fluctuations, as oxygen is more sensitive than carbon to early diagenetic processes (e.g. Swart, 2015). After these considerations, and given the paleoceanographic and paleoclimatic target of this work, we chose to rely on the clearer $\delta^{13}C$ and $\delta^{18}O$ signals obtained from individual genera of foraminifera.

2) Significance of $\delta^{13}C$ and $\delta^{18}O$ data from individual genera of foraminifera

Specimens of foraminifera have been selected from three genera (*Acarinina*, *Subbotina*, and *Cibicidoides*) for stable isotopes analyses. The number of specimens analysed depended on the size of the tests (see methods). These three genera have been selected as representative of three different environments across the water column (surface water, thermocline, and bottom, respectively). Therefore, the isotopic composition of each individual genus depends on the environmental conditions of its specific living environment and, differently from the bulk, is not mixed with carbonate from other sources. However, diagenesis may bias the $\delta^{13}C$ and $\delta^{18}O$ compositions in ancient carbonate microfossils (e.g. Edgar et al., 2015).

SEM imaging of selected specimens representative of the three studied genera of foraminifera reveal a general very good preservation (Fig. S2). However, all the observed specimens display evidence of recrystallization of the test wall, particularly those of *Acarinina* that have smaller and more fragile shells (Fig. S2, e-f). Moreover, it seems that significant infill is present. These features indicate that diagenesis may have altered the primary stable isotope signals. Considering the stable isotopes values

of the foraminifera from the Baskil section, we observe that the average $\delta^{13}C$ of *Cibicidoides* is 0.83‰ lower than *Subbotina*, which is 0.76‰ lower than *Acarinina*

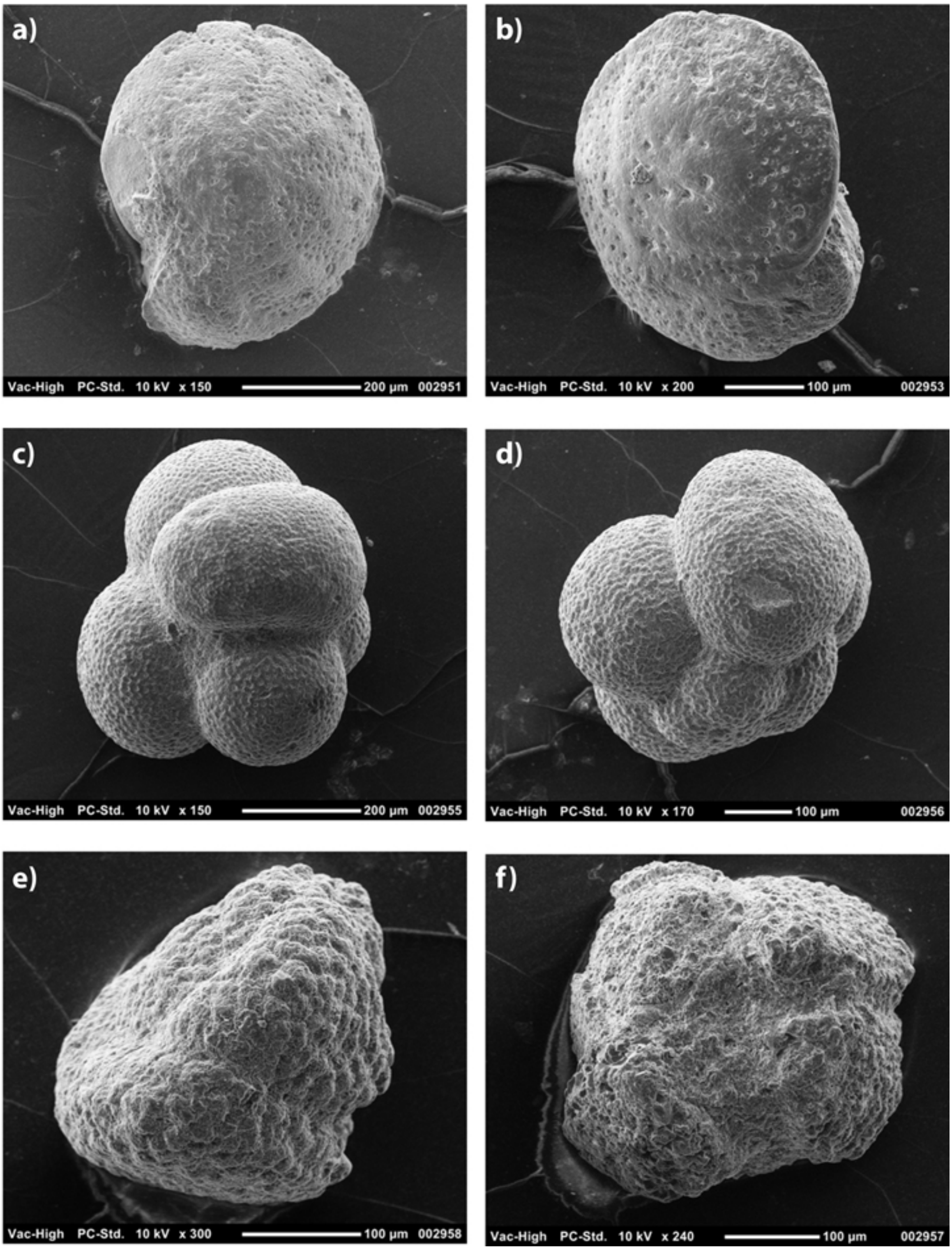

Fig. S2: Representative specimens of *Cibicidoides* (a, b), *Subbotina* (c, d), and *Acarinina* (e, f), from samples T321, T69.5, and T73, of the Baskil section, respectively.

Table 1. Average isotopic differences between foraminiferal genera

		d13C	d18O
Entire section	Subb-Acar	-0.76	0.40
	Cib-Subb	-0.83	1.31
Below 130m	Subb-Acar	-0.79	0.22
	Cib-Subb	0.88	-1.35
Above 130m	Subb-Acar	-0.72	0.60
	Cib-Subb	0.77	-1.27

(Table 1). These offsets are of the same magnitude of those observed in present environments and thus do not seem diagenetically altered to a significant extent. Only in the interval between 40 and 90 m the preservation of *Cibicidoides* is poorer and the $\delta^{13}C$ curve is clearly noisier than the others, indicating a possible diagenetic alteration. The $\delta^{18}O$ offsets between the three genera are also consistent with primary values; however, there is a significant difference in the offset between *Subbotina* and *Acarinina* below and above 130 m (Table 1). This difference is expressed by the $\Delta^{18}O$ parameter and its significance is discussed in the main article.

A comparison between recrystallized and well-preserved foraminifera from the middle Eocene has shown that the formers have $\delta^{18}O$ few permills higher than the latters, whereas the $\delta^{13}C$ is almost the same (Edgar et al., 2015). This supports our observations that most of the $\delta^{13}C$ values of the foraminifera of the Baskil section are not significantly affected by diagenesis. On the other hand, a little diagenetic alteration may explain why the $\delta^{18}O$ values of *Acarinina* are up to 1‰ heavier than of *Subbotina* between 87 m and 130 m. If this signal were primary, it would imply that the temperature was lower at the surface than at the thermocline, which would be very difficult to explain. Considering this evidence and the fact that *Acarinina* is generally more intensely recrystallized than *Subbotina,* we cannot exclude a diagenetic bias also in other parts of the *Acarinina* $\delta^{18}O$ curve. However, as diagenesis tends to increase

the $\delta^{18}O$ values, it would reduce the offset between the curves of *Acarinina* and *Subbotina*. This means that if the $\delta^{18}O$ of *Acarinina* were diagenetically altered then the original $\Delta^{18}O$ between *Acarinina* and *Subbotina* would have been even larger, and thus it would even better support our interpretation. In general, we observe that diagenesis does not significantly affect most of the stable isotopes values of the foraminifera of the Baskil section, but we cannot completely exclude a diagenetic influence. For being conservative with our interpretation, we considered only the curves smoothed by 3 points moving average and variations represented by several data points and of 0.5‰ amplitude or larger. We also stress that absolute values are probably not always representative of the primary parameters, particularly for $\delta^{18}O$ of *Acarinina*, however, the trends can be confidently interpreted.

Another possible bias related to stable isotopes in foraminifera is vital effect. This occurs when, for some reasons, the carbonate of a specific fossil shell does not precipitate in isotopic equilibrium with the environment (i.e. Sharp, 2007). We tried to minimize the vital effect by measuring multiple specimens per sample (3 to 5 for *Cibicidoides* and *Subbotina*, 10 to 15 for *Acarinina*, which is significantly smaller) and, as mentioned before, by basing our interpretation only on curves smoothed by 3 points moving average and on variations of 0.5‰ amplitude or larger.

3) Paleoceanographic and paleoecological changes during the MECO

The stable isotopes shifts that identify the MECO in the Baskil section are accompanied by important changes in calcareous nannofossils assemblages (Fig. S3). Various groups of calcareous nannofossils increase during or soon after the MECO, whereas others decrease and some disappear (Fig. S3). Despite the paleoecological interpretation of several groups of nannofossils is still controversial, we can infer that

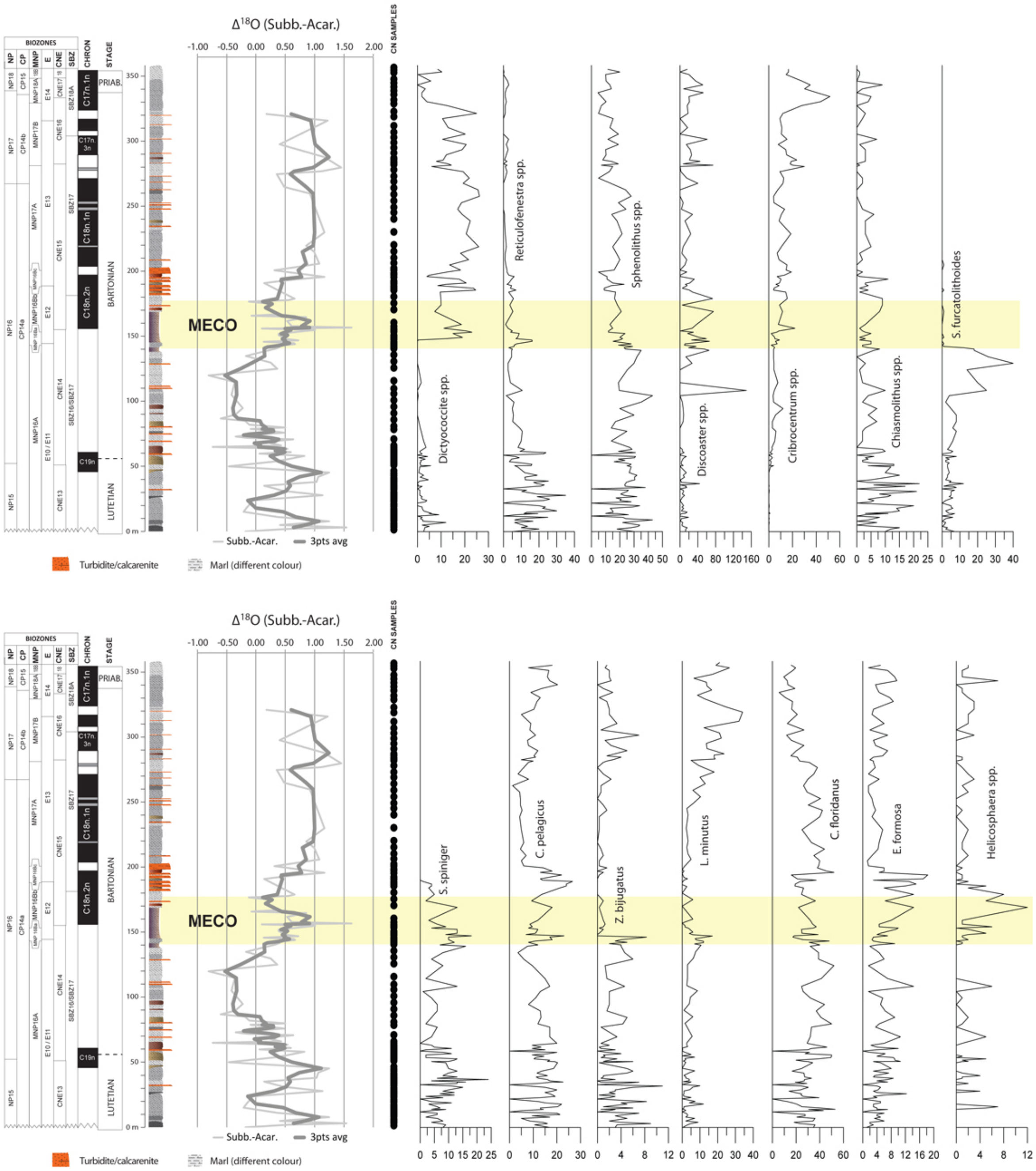

Fig. S3: Δ^{18}O and calcareous nannofossils abundances across the Baskil section

the abrupt increase in the abundance of *Dictyococcites, Ericsonia formosa,* and *Helicosphaera* during the MECO can be related to a shift towards warmer and more eutrophic conditions. This agrees with mineralogical and geochemical data from Rego et al. (2018) and shown in the figure 3 of the main article, which display calcite decrease, detrital mineral increase, and relative increase in smectite and decrease in

illite accompanying the increase in $\Delta^{18}O$. As discussed in the main text and in Rego et al. (2018), this suggests the onset of warm and humid conditions with prevailing chemical weathering at the MECO, which increased the runoff from the land, freshened the sea surface, and then enhanced the density contrast between the surface and the thermocline.

References

Edgar, K.M., Anagnostou, E., Pearson, P.N. and Foster, G.L., 2015. Assessing the impact of diagenesis on δ11B, δ13C, δ18O, Sr/Ca and B/Ca values in fossil planktic foraminiferal calcite. *Geochimica et Cosmochimica Acta*, *166*, pp.189-209.

Hudson, J.D., 1977. Stable isotopes and limestone lithification. *Journal of the Geological Society*, *133*(6), pp.637-660.

Rego, E.S., Jovane, L., Hein, J.R., Sant'Anna, L.G., Giorgioni, M., Rodelli, D. and Özcan, E., 2018. Mineralogical evidence for warm and dry climatic conditions in the Neo-Tethys (eastern Turkey) during the middle Eocene. *Palaeogeography, Palaeoclimatology, Palaeoecology*, *501*, pp.45-57.

Rodelli, D., Jovane, L., Özcan, E., Giorgioni, M., Coccioni, R., Frontalini, F., Rego, E.S., Brogi, A., Catanzariti, R., Less, G. and Rostami, M.A., 2018. High-resolution integrated magnetobiostratigraphy of a new middle Eocene section from the Neotethys (Elazığ Basin, eastern Turkey). *GSA Bulletin*, *130*(1-2), pp.193-207.

Sharp, Z., 2007. Principles of stable isotope geochemistry: Pearson Prentice Hall. *New Jersey, 344p.*

Swart, P.K., 2015. The geochemistry of carbonate diagenesis: The past, present and future. *Sedimentology*, *62*(5), pp.1233-1304.
